# Cellular and Noncellular Approaches for Repairing the Damaged Blood–CNS–Barrier in Amyotrophic Lateral Sclerosis

**DOI:** 10.3390/cells13050435

**Published:** 2024-02-29

**Authors:** Larai Manora, Cesario V. Borlongan, Svitlana Garbuzova-Davis

**Affiliations:** 1 Center of Excellence for Aging & Brain Repair, Morsani College of Medicine, University of South Florida, 12901 Bruce B. Downs Blvd., MDC 78, Tampa, FL 33612, USA; laraimanora@usf.edu (L.M.); cborlong@usf.edu (C.V.B.); 2 Department of Neurosurgery and Brain Repair, Morsani College of Medicine, University of South Florida, 12901 Bruce B. Downs Blvd., MDC 78, Tampa, FL 33612, USA

**Keywords:** ALS, G93A SOD1 mice, blood–CNS–barrier, stem cell transplantation, nanoparticles, repair

## Abstract

Numerous reports have demonstrated the breakdown of the blood–CNS barrier (B-CNS-B) in amyotrophic lateral sclerosis (ALS), a fatal neurodegenerative disease. Re-establishing barrier integrity in the CNS is critical to prevent further motor neuron degeneration from harmful components in systemic circulation. Potential therapeutic strategies for repairing the B-CNS-B may be achieved by the replacement of damaged endothelial cells (ECs) via stem cell administration or enhancement of endogenous EC survival through the delivery of bioactive particles secreted by stem cells. These cellular and noncellular approaches are thoroughly discussed in the present review. Specific attention is given to certain stem cell types for EC replacement. Also, various nanoparticles secreted by stem cells as well as other biomolecules are elucidated as promising agents for endogenous EC repair. Although the noted in vitro and in vivo studies show the feasibility of the proposed therapeutic approaches to the repair of the B-CNS-B in ALS, further investigation is needed prior to clinical transition.

## 1. Introduction

**Figure 1:**
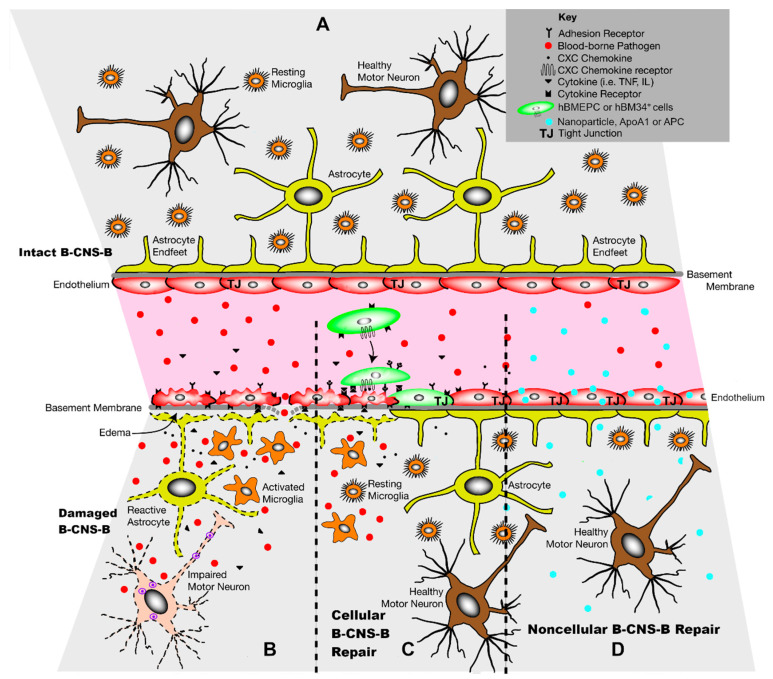
Schematic representation of cellular and noncellular treatment approaches to the repair of damaged B-CNS-B in ALS. (**A**) The intact B-CNS-B is composed of a single EC layer and other elements (pericytes, basement membrane, tight/adherens junctions, and perivascular astrocytes) and controls CNS homeostasis through the selective transport of molecules and cells through the capillary endothelium. (**B**) In ALS, damaged leaky endothelium is key to CNS barrier impairment, allowing for the entry of harmful blood-borne substances, which might accelerate motor neuron degeneration. In addition, increased perivascular edema, activated microglia, reactive astrocytes, and various chemokine and pro-inflammatory cytokine secretions could be exacerbated by degenerated endothelium during disease progression. (**C**) Replacement of damaged ECs via administration of hBMEPCs or hBM34+ cells (arrow) may restore barrier integrity and retard motor neuron degeneration. Recruitment of transplanted cells to sites of endothelium damage is likely aided by “signaling” molecules released from degraded ECs. (**D**) For enhancing endogenous EC repair, systemic delivery of nanoparticles (EVs or exosomes), ApoA1, or APC may exert protective effects by entering into damaged ECs and releasing various biomolecules. Restoring EC integrity may lead to B-CNS-B repair and result in increased motor neuron survival. Purple shading indicates blood in capillary lumen. Abbreviations: hBMEPC—human bone marrow-derived endothelial progenitor cells. hBM34^+^ cells—human bone marrow-derived CD34 positive cells, ApoA1—apolipoprotein A1, APC—activated protein C. Note: significant modifications were made in this figure which was adapted from our previously published figure (Garbuzova-Davis et al. [15]).

Amyotrophic lateral sclerosis (ALS) is a progressive neurodegenerative disease characterized by widespread motor neuron degeneration in the brain and spinal cord. ALS is a multifactorial fatal disorder with a complex pathology [1,2,3,4], making it challenging to generate effective therapies to slow or retard disease progression and resulting in the limited number of therapeutic options currently available. One potential factor contributing to ALS pathogenesis is an altered blood–CNS–barrier (B-CNS-B), composed of the blood–brain barrier (BBB) and the blood–spinal cord barrier (BSCB). Degenerated capillary endothelial cells (ECs), impairment of endothelial transport system, damaged mitochondria in EC and neuropil, reduced pericyte coverage, altered astrocyte end-feet processes, downregulated tight junction (TJ) protein expressions, leaky microvasculature, and perivascular edema were discovered in brains and spinal cords of animal models of disease [5,6,7,8,9,10] and ALS patients [11,12,13,14]. Importantly, BBB and BSCB breakdown was found in SOD1 mutant mice and rats prior to motor neuron degeneration and neurovascular inflammation [7,8,10]. The authors showed a reduction of TJ proteins (ZO-1, occludin, and claudin-5), detection of microhemorrhages with the release of neurotoxic hemoglobin-derived products (hemosiderin), and IgG leakage in the pre-symptomatic stage of SOD1 mutant animals, suggesting vascular alterations as an early ALS pathological event. These accumulated microvascular pathologies indicate a dysfunctional B-CNS-B, leading to the entry of harmful substances, including immune/inflammatory cells, from the peripheral blood circulation into the CNS parenchyma and potentially exacerbating motor neuron degeneration in ALS (Figure 1B). 

Comprehensive reviews [16,17] have discussed not only the mechanisms underlying B-CNS-B impairment and translational implications of barrier dysfunction in ALS, but also potential strategies for restoring barrier integrity. Mirian et al. [17] emphasized that B-CNS-B “structural and functional abnormalities are likely implicated in ALS pathophysiology and may occur upstream to neurodegeneration”. Also, the authors noted the necessity for developing therapeutic strategies targeting B-CNS-B dysfunction in ALS. 

Stem cell therapy for ALS is primarily advanced for the replacement of degenerated motor neurons or improvement of the CNS microenvironment to delay and/or prevent motor neuron death (reviewed in [18,19,20,21,22,23,24]). However, due to multiple locations of motor neurons in segmented spinal cord and the complexity of neuronal interconnectivity, motor neuron replacement is not achievable. A more practical approach would be engineered stem cells to secrete growth factors for motor neuron survival or to combine stem cells with specific trophic factors for modulation of the CNS microenvironment to rescue dying motor neurons [25,26,27]. Thus, neuroprotection is a more feasible treatment goal than neural replacement. 

One potential therapeutic strategy for the protection of motor neurons in ALS is the repair of the damaged B-CNS-B by preventing detrimental peripheral blood substances from entering the CNS. Barrier restoration may be achieved *directly* through the cellular replacement of damaged ECs in the capillary lumen or *indirectly* via the cellular secretion of numerous molecules for enhancing endogenous EC survival. Various stem cell types and small bioactive particles, which are secreted by stem cells and may exert protective effects by their interaction with damaged cells (reviewed in [28]), are potential contenders for engendering EC repair. For the replacement of damaged ECs in ALS, stem cells with the capability to differentiate into numerous cell types [23,29], including into cells of endothelial lineage [30,31,32,33], can be pursued. An attractive candidate in therapeutic intervention for improving EC survival in ALS is the extracellular vesicle (EV) secreted by stem cells. Importantly, EVs mediate communication between cells by transferring various peptides, proteins, lipids, mRNA, and microRNA to recipient cells, thus facilitating diverse biological processes under healthy and diseased physiological conditions (see reviews in [34,35,36,37,38,39]). Since EVs contain various bioactive vesicular cargoes, these nanoparticles may serve as cell-free therapeutic agents maintaining EC functionality and leading to endothelium repair in ALS [40] or may have “potential use as therapeutic delivery system” [28]. Relatively recently, specific focus has been directed to the construction of specialized nanovesicles via bioengineering with certain molecules [41,42,43] that can target ECs. Also, this review elucidates other biomolecules such as Apolipoprotein A1 (ApoA1) and activated protein C (APC) for the potential restoration of barrier integrity in the CNS.

Here, proposed cellular and noncellular approaches to B-CNS-B repair in ALS are discussed in detail. Additionally, advantages and limitations of recommended therapeutic interventions are noted. 

## 2. Cellular Approach to B-CNS-B Repair

### 2.1. The Effects of Bone Marrow-Derived Stem Cells

The restoration of the altered B-CNS-B is the main goal in treating ALS and can be achieved by the replacement of damaged ECs via cell administration. Bone marrow is a primary source of hematopoietic stem cells and potentially includes the putative endothelial progenitor cells (EPCs) [31,32,33,44]. Although CD34^+^ cells are pluripotent hematopoietic stem cells and capable of differentiating into multiple hematopoietic cell lineages [45], EPCs have been shown to be rich in CD34^+^/CD45^−^ cell populations [46]. In terms of determining whether CD34^+^ cells are a promising source of cells for B-CNS-B restoration, human bone marrow CD34^+^ (hBM34^+^) cells were intravenously (iv) transplanted at three different doses of 5 × 10^4^, 5 × 10^5^, and 1 × 10^6^ cells into a symptomatic G93A SOD1 murine model of ALS [47]. The study results demonstrated that the mice, mainly those receiving the highest cell dose, better retained motor function through enhanced motor neuron survival. Importantly, the engraftment of transplanted cells was noted in numerous capillaries of the spinal cord, and these cells showed differentiation into ECs. Also, reduced astro- and microgliosis, maintained perivascular astrocyte end-feet processes, and decreased parenchymal permeability for Evans blue (EB) dye were determined in spinal cords of ALS mice predominantly treated with the high dose of 1 × 10^6^ hBM34^+^ cells. Also, these post-transplanted mice exhibited improvements in ultrastructural capillary morphology, identified through electron microscopy [48], in addition to a significant reduction of spinal cord microhemorrhages [49]. Together, the noted benefits of transplanting bone marrow hematopoietic stem cells into symptomatic ALS mice may support optimal doses of these cells for the potential repair of barrier integrity. However, a large number of severely damaged capillaries in the cervical and lumbar spinal cords were detected via ultrastructural analysis even after transplanting a high dose of hBM34^+^ cells. Also, some transplanted cells, expressing hematopoietic common leukocyte CD45 antigen, were found not only within capillary lumen but also distant from blood vessels in the spinal cords, likely a sign of “differentiation of transplanted cells into cells with different immunophenotypes” [47]. Potentially, the administration of cells with restricted endothelial cell lineage would be a superior strategy for B-CNS-B restoration in ALS.

To determine the effectiveness of human bone marrow-derived endothelial progenitor cells (hBMEPCs) as a cell source for CNS barrier restoration, hBMEPCs were characterized in vitro at various times under normogenic conditions [50]. Cultured cells showed well-defined morphologies with the re-arrangement of cytoskeletal F-actin filaments and positive immunoexpression for CD105, indicating the EC phenotype. Also, gradually increased VEGF-A and angiogenin-1 levels were detected in conditioned media via ELISA. In addition, immunoexpressions for ZO-1 and occludin were found on cell membranes of adjacent hBMEPCs. Thus, these in vitro results, demonstrating secretion of angiogenic factors and TJ protein expressions, indicate the potential utility of hBMEPCs for the repair of the damaged B-CNS-B in ALS. However, this possibility needs in vivo confirmation.

In a follow-up study, the effects of hBMEPC transplantation into symptomatic G93A SOD1 mice at a 1 × 10^6^ cell dose were evaluated at weeks post transplant [51]. The study results primarily revealed that hBMEPC treatment substantially ameliorated disease behavioral outcomes, leading to delayed progression of disease at least for 2 weeks compared to media-injected ALS mice. Transplanted cells engrafted into capillaries of gray and white matter spinal cord as well as brain motor cortex and brainstem. Importantly, administered hBMEPCs were exclusively detected within the capillary wall and were not identified outside the capillary lumen. Also, restored capillary ultrastructure and reduced EB extravasation into the spinal cord parenchyma were found in cell-treated mice. Notably, motor neuron survival was enhanced in the spinal cord, and the integrity of perivascular astrocyte end-feet was re-established. These study results showed the effectiveness of hBMEPCs towards restoring barrier integrity. Moreover, the detection of human DNA via RT-PCR assay in isolated mouse CNS ECs demonstrated greater levels in mice receiving hBMEPCs vs. hBM34^+^ cells [52], confirming human cell engraftment in murine capillaries. 

In general, intravenously administered hBM34^+^ cells and hBMEPCs into symptomatic G93A SOD1 mutant mice revealed beneficial effects on the B-CNS-B reparative processes. However, the impacts of cell transplantation on the integrity of the endothelium in murine CNS capillaries were not fully established. In a study by Garbuzova-Davis et al. [53], functional and cellular constituents of the microvascular endothelium in the spinal cord were evaluated after the administration of hBM34^+^ cells and hBMEPCs, at the same dose of 1 × 10^6^ cells, into G93A SOD1 mice. The findings showed that ALS mice receiving hBMEPC vs. hBM34^+^ cell treatment significantly increased the levels of TJ claudin-5, occludin, and ZO-1 proteins; enhanced the coverage of capillary pericytes; amended immunoexpression of basement membrane laminin; and increased expression of endothelial cytoskeletal F-actin. These study results offered significant proof that treatment with a specific human bone marrow-derived cell type such as hBMEPCs might possibly lead to BSCB repair in ALS. 

The studies described above have shown the benefits of transplanted stem cells derived from human bone marrow on B-CNS-B restoration during disease progression, and long-term post-transplant cell effects have recently been revealed [54]. It has been shown that G93A SOD1 mice receiving hBMEPC vs. hBM34^+^ cell transplants improved the amelioration of behavioral outcomes until near disease end-stage and significantly increased lifespan vs. media mice. About 36% of hBMEPC-treated mice were still alive at the age of 20 weeks vs. 20% of hBM34^+^-treated animals. No media ALS mice survived until 20 weeks. These findings highlighted that hBMEPCs prolonged the behavioral function and increased the ALS mouse’s survival, likely due to BSCB repair. However, the lifespan of hBMEPC-treated mice was only modestly increased, and repeated cell transplants may be required for improving barrier endothelium integrity during disease progression. This suggestion should be addressed in future investigations. 

Together, numerous reports evidence that transplanted stem cells derived from a restricted cell lineage, as endothelial progenitor cells, better improve B-CNS-B restoration in a murine model of ALS vs. non-differentiated hBM34^+^ cells. Figure 1C demonstrates the potential mechanism of damaged EC replacement by the recruitment of administered cells to sites of injury via “signaling” molecules released from degenerated ECs. Barrier restoration via a cellular approach, from a translational viewpoint, has demonstrated therapeutic advantages when initiated upon the appearance of disease symptoms.

### 2.2. The Effects of Mesenchymal Stem Cells

Mesenchymal stem cells (MSCs) are multipotent and may differentiate into various cell types (reviewed in [23,29,55,56,57]). MSCs can be derived from different tissues such as bone marrow or adipose tissues. Numerous studies have demonstrated the therapeutic efficacy of MSCs using SOD1-mutant mice [25,58,59,60,61,62]. The benefits of the MSC treatment of ALS mice have been shown through sustained motor function, delayed motor neuron degeneration, and extended animal survival, which are mainly achieved by the secretion of various neurotrophic factors (GDNF, VEGF, and bFGF). Moreover, clinical trials were conducted on allogeneic or autologous MSC transplantation via intraspinal, intrathecal, or intracerebral administration into ALS patients, and they showed feasibility in treatment without major adverse effects [60,63,64]. Nonetheless, no functional improvement in patients was determined. 

However, there are limited reports addressing whether MSCs ameliorate the B-CNS-B status in ALS. So far, one study, by Magota et al. [59], has investigated potential BSCB repair in G93A SOD1 transgenic female rats through the injection of 1 × 10^6^ MSCs via the femoral vein. Notably, MSCs were obtained from the bone marrow of adult wildtype rats, and phenotypic cell analysis was conducted prior to cell administration. The results showed that MSC treatment reduced the vascular leakage of Evans blue dye, increased the length of ECs and pericytes in capillaries, and increased the expression of neurturin neurotrophic factor in the lumbar spinal cord 2 weeks post injection. Treated rats also better maintained behavioral hind limb function and reduced motor neuron loss. The authors concluded that observed BSCB restoration led to the inhibition of motor function deterioration as determined by forelimb–hindlimb coordination (BBB scoring scale) and rotarod tests in ALS rats. 

Thus, MSCs might be a promising source of cells for B-CNS-B repair in ALS. However, more prolonged effects of MSCs treatment on barrier status need to be shown. Also, the specific MSC-differentiated cell type(s) that contribute to neuroprotection require confirmation in vivo. 

## 3. Noncellular Approach to B-CNS-B Repair

### 3.1. The Effects of Extracellular Vesicles Derived from Stem Cells 

Extracellular vesicles (EVs) are a heterogeneous group of nanovesicles composed of microvesicles (MVs), exosomes, and apoptotic bodies, that are released by numerous cell sources, including stem cells (SCs) (reviewed in [65,66,67,68]). Various biomolecules encapsulated in EVs, surrounded by a lipid bilayer, play a crucial role in cell-to-cell communication. Stem cell-derived EVs may be useful in preserving diseased tissue by increasing angiogenesis, reducing inflammation, and delivering various factors within and outside of the CNS [69]. 

Stem cells, with their ability to repair damaged tissue, are an important area of research in the regenerative medicine field. The effectiveness of stem cell therapies may be based on cells’ capacity to release nanoparticles such as EVs. The EVs, endogenously released by SCs or administered through various routes to the body, interact with other cell types and could provide beneficial effects via the secretion of various biomolecules. Deregibus et al. [70] showed that MVs isolated from EPC-derived peripheral blood mononuclear cells were incorporated into microvascular ECs in vitro, promoting cell proliferation and survival. Also, the authors showed that these MVs improved capillary formation and increased angiogenesis in severe combined immunodeficient mice through the transfer of mRNA that promotes PI3K/AKT signaling pathway and suggested that MVs are able to trigger an angiogenic program. In another study [71], CD34^+^ SC-derived exosomes also showed angiogenic potential by increasing capillary density and expressing vascular endothelial growth factor-1 (VEGF), angiogenin-1 (ANG1), and matrix metallopeptidase-9 (MMP9) in mouse ischemic hindlimb tissue via upregulated proangiogenic microRNA expression. In a lung ischemia-reperfusion injury model, MSC-derived-EVs reduced pulmonary edema and neutrophil migration across the endothelial cells [72]. These EVs also decreased EC barrier permeability at least partly through modulating immune cell activation and releasing anti-inflammatory molecules (prostaglandin E2, keratinocyte growth factor, and IL-10). Additionally, human adipose MSC-derived exosomes internalized by electroporated rat corneal ECs demonstrated the inhibition of EC autophagy and mitochondrial dysfunction, promoting EC recovery and proliferation after cryoinjury, leading to EC regeneration [73]. Altogether, these study results indicate that SC-derived EVs can significantly improve EC function and recovery after injury in several diseases, pointing to EVs’ potential for enhancing B-CNS-B integrity in ALS. 

While research on the effects of EVs in promoting B-CNS-B integrity is limited, several studies have shown that EVs can directly act on the BBB and BSCB. Using exosomes from endothelial colony-forming cells, a population of early-lineage EPCs in human umbilical cord blood, Gao et al. [74] demonstrated that these exosomes delivered intravenously (iv) into mice with traumatic brain injury (TBI) produced a significant, but modest, decrease in brain edema and Evans blue (EB) leakage in the BBB of post-TBI mice. Interestingly, following tPA-induced BBB disruption, MSC-derived EVs administered intravenously reduced cerebral hemorrhage and EB extravasation, leading to decreased neurological deficits in a mouse model of ischemic stroke [75]. In a rat model of spinal cord injury, intravenously administered MSC-derived EVs significantly improved locomotor function and attenuated death of spinal cord neurons [76]. These beneficial EV effects were accompanied by reduced EB extravasation and increased pericyte capillary coverage, improving BSCB function. These studies demonstrate that MSC-EVs restored BBB and BSCB integrities; however, direct EV interactions with damaged ECs require further study. Recently, the intravenous administration of MSC-EVs derived from human umbilical cord into SCI female rats were determined to promote neurological function recovery and mitigate BSCB disruption by reducing barrier permeability for EB and FITC-dextran via the downregulation of endothelin-1 [77]. Importantly, Western blot analysis also showed that EV treatment increased TJ ZO-1, β-catenin, occluding, and claudin-5 protein expressions in the spinal cord. Overall, current proposed mechanisms for EVs’ beneficial effects on B-CNS-B repair include downregulating endothelin-1 expression, downregulating NF-kB p65, activating AKT signaling to prevent cell death, and downregulating TLR4/NF-kB expression to reduce pro-inflammatory cytokine release, respectively [74,75,76,77]. While these mechanisms suggest promising outcomes for EV treatment of damaged B-CNS-B, the effects of SC-derived EV treatment for barrier repair in ALS have not been fully elucidated. 

To evaluate the effects of EVs on ECs, Garbuzova-Davis et al. [40] isolated EVs from conditioned media of cultured hBMEPCs, which were then co-cultured with mouse brain endothelial cells (mBECs). Cultured mBECs subjected to 3% plasma from symptomatic G93A SOD1 mice showed increased cell death, including abnormal cellular morphology, and adding hBMEPC-derived EVs significantly improved mBEC survival. Further examination with fluorescently GFP-labeled hBMEPC-derived EVs showed that these vesicles were taken up by mBECs in pathologic conditions, and EV cellular entry was inhibited by anti-CD29 blocking antibody, resulting in the increase in EC cell death rates. The most significant reduction in mBEC death following ALS plasma exposure and hBMEPC-derived EV treatment was found when 1 µg/mL of EVs was added to cultured cells. These results demonstrated that EVs from SCs ameliorated cell death in an ALS-like environment in vitro but in a dose-dependent fashion, as adding 5 µg/mL of EVs to mBECs resulted in increased cell death [40]. Following this significant finding that hBMEPC-EVs are taken up by ECs and prevent cell death in ALS-like pathologic conditions, testing the efficacy of this non-cellular approach in ALS in vivo is imperative. Overall, SC-derived EVs show promise for strategies to restore B-CNS-B integrity and may lead to a treatment for ALS. Based on these results, SC-derived EVs could prove even more practical in treating ALS patients than SC transplant therapy *per se* due to EVs’ small size, potential for reduced immunoreactivity, and endogenous effects on existing ECs by promoting angiogenesis and resistance to apoptosis. However, these EVs have disadvantages that call for a continued investigation of their therapeutic potential and ways to fine-tune beneficial effects. Specifically, EVs appear to exert their beneficial effects in a bell-curve fashion related to dosage [40,76]. Interestingly, hBMEPC-EVs increased EC death to similar levels as 3% ALS plasma when these cells were treated with a concentration of 5 µg/mL, showing that high EV concentrations may be toxic to ECs and calling for careful determination of therapeutic doses in ALS patients [40]. Additionally, Bonafede et al. [78] investigated the long-term effect of adipose MSC-derived exosome treatment in G93A SOD1 mice and showed beneficial effects from repeatedly administered exosomes, intravenously or intranasally, on motor function, preservation of lumbar motor neurons, neuromuscular junction, and decreased glial cell activation. However, exosome enhancement waned after mice reached 17 weeks of age. This study likely indicates that the continued administration of nanoparticles is necessary for therapeutic results, but EVs are ineffective in delaying progression in late-stage disease. Altogether, further in vivo research on types of SC-derived EVs and their actions during disease progression, including effects on mouse lifespan, is needed prior to clinical efforts at restoring the B-CNS-B in ALS. Also, the proteomic analysis of EV content should be conducted to determine the most beneficial therapeutic biomolecules.

### 3.2. The Effects of Bioengineered EVs 

A potential strategy to improve the efficacy of EVs is to bioengineer them with certain molecules, creating specialized EVs that can be used to target EC dysfunction [43]. For example, hMSCs engineered to contain iron oxide nanoparticles (INOPs) can be serially extruded to produce MSC-exosome mimics called iron oxide nanoparticle-incorporated exosome-mimetic nanovesicles (NV-IONPs); NV-IONPs can be magnet-guided to a specific injury site, minimizing the rate of MSC-exosome entrapment in non-target tissues [41,42]. When NV-IONPs were administered intravenously and guided to the injured spinal cord (SC), many of these nanovesicles reached the site of injury and were found in negligible numbers in other tissues. In the injured SC, NV-IONPs resulted in increased behavioral recovery and reduced neuroinflammation in an SCI mouse model. While researchers did not assess BSCB permeability or whether NV-IONPs were incorporated into ECs of the spinal cord, Kim et al. [42] showed that adding NV-IONPs to human umbilical cord vein ECs increased EC angiogenesis, proliferation, and migration in vitro. Subsequently, in an SCI mouse model, intravenously delivered NV-IONPs increased VEGF, ANG-1, and bone-derived neurotrophic factor (BDNF) expression in the SC and improved behavioral outcomes [42]. But assessing NV-IONPs’ effects on B-CNS-B permeability is a necessary follow-up to these results to test their potential for addressing EC dysfunction. 

In another study, Li et al. [79] also modified bone marrow-derived MSCs through transfection with lentivirus-encoded chemokine receptor type 4 (CXCR4), a receptor for stromal cell-derived factor 1/CXCL12. This receptor is found on ECs and many other cell types, promoting endothelial barrier protection in atherosclerosis, playing a role in angiogenesis, and homing MSCs to sites of injury [80,81,82]. The authors [79] found that CXCR4-overexpressing-MSC-derived exosomes enhanced the proliferation of ECs and increased angiogenesis vs. control MSC-derived exosomes in vitro. In an ischemic stroke rat model, CXCR4-overexpressing-MSC exosomes injected into the lateral ventricle led to decreased neurological deficits and infarct volume in rats to a greater extent than control MSC exosomes [79]. Recently, Rincon-Benavides et al. [83] demonstrated that human dermal fibroblasts, transfected with human-ETV2, -FLI1, and -FOXC2 plasmids, released EVs, called EFF EVs, and induced the conversion of somatic cells into ECs. These EFF EVs containing angiogenic factors such as VEGF and fibroblast growth factor 2 have better promoted angiogenesis and reduced wound areas in immunodeficient mice than control EVs [83]. While these studies with NV-IONPs, CXRC4-overexpressing-MSC exosomes, and EFF EVs provided important evidence that engineered EVs may lead to better outcomes than typical SC-derived EVs, the direct effects of these molecules on damaged endothelium are yet unknown. To demonstrate the effectiveness of engineered EVs in targeting the dysfunctional BSCB, Xie et al. [84] engineered MSC exosomes derived from CD146+CD271+ human umbilical cord MSC subpopulations to express the Arg-Gly-Asp (RGD) peptide. RGD-CD146+CD271+ exosomes delivered intranasally reduced entry of EB into surrounding tissue, increased TJ expression, and promoted neurological recovery in post-SCI mice. Together, these results signify that engineered EVs may be promising options for advancing B-CNS-B repair in ALS. However, more research comparing engineered EVs and natural SC-derived EVs is necessary. 

### 3.3. Addressing B-CNS-B Repair in ALS with ApoA1 

There are other emerging non-cellular strategies with potential therapeutic options for B-CNS-B restoration in ALS. One such option is Apolipoprotein A1 (ApoA1), a major constituent of HDL that highly promotes the cellular efflux of lipids from tissues and carries lipids to the liver for cholesterol recycling. While some studies are contradictory concerning the effects of lipid metabolism in ALS, patients experience an imbalance in LDL/HDL and ApoB/ApoA1 levels that has been linked to pathologic hyperlipidemic, atherosclerotic, and pro-inflammatory EC damage in ALS [85,86,87]. Recently, a longitudinal study revealed that higher HDL and ApoA1 levels were associated with decreased ALS risk [88], but additional studies should investigate the potential mechanism underlying ALS prevention or recovery with ApoA1 and determine ApoA1’s actions in CNS endothelium repair.

Notably, Garbuzova-Davis et al. [89] investigated the therapeutic potential of ApoA1 on mBEC status in ALS-like conditions in vitro. The results showed that adding ApoA1 to culture media in a dose-dependent manner protected mBECs from cell injury induced by exposure to plasma from symptomatic G93A SOD1 mice. The greatest reduction in cell death was found at ApoA1 concentration of 100 µg/mL. Also, co-culturing mBECs and hBMEPCs in ALS mouse plasma demonstrated that hBMEPCs secreted ApoA1, which integrated into mouse ECs via an activated PI3/Akt signaling pathway to exert beneficial effects [89]. These initial results suggest that ApoA1 is a promising agent for B-CNS-B repair, but the authors noted that ECs were cultured in fetal bovine serum, which may have influenced the lipid content of ECs. Further investigation into ApoA1’s mechanisms for repair could be conducted with ECs cultured in serum-free media to control this variable and determine the effects of ApoA1 on dysfunctional ECs [89]. Also, in vivo research on ApoA1’s actions at damaged CNS endothelium in ALS is needed to confirm whether the proposed treatment leads to B-CNS-B restoration. Currently, the effects of ApoA1 administration into symptomatic G93A SOD1 mice of both genders are under investigation, supported by an NIH grant (1R21NS132576-01).

### 3.4. Addressing B-CNS-B Repair in ALS with Activated Protein C 

Among the numerous factors involved in ALS pathogenesis, neuroinflammation is a major contributor to disease progression, potentially leading to B-CNS-B breakdown. To address this issue, a multi-target treatment might be required. One potential candidate for such intervention is activated protein C (APC), an endogenous plasma protease with several benefits in combating CNS injuries and disorders as an anti-inflammatory, anti-thrombotic, and cytoprotective agent [90,91,92,93]. Due to its multiple beneficial actions, APC has been proposed as a disease-modifying therapeutic intervention (reviewed in [94,95]). Also, APC may be involved in neuroprotection not only by controlling neuroinflammation, but also in the stabilization of the B-CNS-B. APC is able to cross the BBB and beneficially concentrate in the CNS, and the endothelial protein C receptor mediates this passage [96]. Zhong et al. [97] showed that intraperitoneal injections of APC analogs into symptomatic G93A SOD1 mice ameliorated disease progression and increased lifespan. Moreover, APC postponed microglia activation and decreased serum protein IgG and hemoglobin-derived product leakage across the BSCB. Additionally, TJ ZO-1 and occludin protein expressions were restored in spinal cord capillaries of APC-treated mice. The authors noted that APC entering into the CNS of ALS mice acts exclusively on motor neurons and microglia “to directly inhibit disease progression by reducing mutant SOD1 transcription” [97]. Although APC may have beneficial effects in ALS treatment, it is unclear whether this protective agent’s effects on the damaged endothelium occur independently or as result of downregulating inflammatory response by glial cells. Additional studies are needed to elucidate this issue. 

Together, proposed noncellular treatments may enhance endogenous EC repair through the entry of various biomolecules into the damaged endothelium, leading to B-CNS-B restoration and motor neuron survival in ALS as schematically illustrated in Figure 1D. 

## 4. Conclusions

Repairing the impaired B-CNS-B in ALS is essential to prevent further motor neuron degeneration from harmful substances in the systemic circulation. Treatment options for barrier restoration may be achieved via cellular or noncellular approaches, which have been discussed in detail. Table 1 highlights relevant studies, listing advantages and limitations of the proposed treatments. Also, Figure 1 shows the applicability of the different therapeutic methodologies for restoring the CNS barrier status in ALS. For the cellular repair of the damaged B-CNS-B, stem cells derived from bone marrow may be pursued for therapy via the direct replacement of damaged ECs. As emphasized, stem cells derived from a restricted cell lineage such as endothelial progenitor cells transplanted into symptomatic ALS mice exclusively adhered to the capillary lumen and significantly improved structural and functional B-CNS-B integrity. In clinical settings, bone marrow-derived endothelial cells may be used as an autologous or allogenic cell source. For noncellular intervention with the goal of enhancing endogenous EC survival, extracellular vesicles or exosomes secreted by stem cells may exert protective effects by interacting with damaged cells via the delivery of various biomolecules, thereby improving the motor neuron environment and preventing motor neuron degeneration. The bioengineering of nanovesicles with certain molecules may be an appealing strategy for advancing barrier repair in ALS. Specialized nanoparticles can be created to target EC dysfunction. However, prior to this application, studies on the molecular profile within degenerated ECs should be conducted. Also, other biomolecules such as ApoA1, a major constituent of HDL, and APC with cytoprotective and anti-inflammatory actions may be pursued for the restoration of barrier integrity in the CNS. Thus, although a cell-based therapy for the restoration of the damaged B-CNS-B in ALS can be approached, noncellular methodologies may be more beneficial as therapeutics and could be combined with traditional ALS treatment such as riluzole or edavarone. Additionally, systemic administration is the preferable route for cellular or noncellular particles to restore altered microvascularity in ALS patients due to the widespread distribution of transplanted cells or desired particles within the CNS. However, some administered cells or agents may reside outside the CNS, a potential disadvantage of intravenous transport. To address this issue, dose–response studies will be necessary. Also, repeated administrations can be advanced due to worsening B-CNS-B status during disease progression. Nevertheless, despite the fact that various in vitro and in vivo studies have demonstrated the feasibility of therapeutic strategies for the repair of the CNS barriers in ALS, more investigations on preferable stem cell type or nanoparticles, including their route of administration should be pursued before clinical applications. Additionally, most effective bioactive noncellular agents for repairing the damaged B-CNS-B in ALS would require pharmacological grading prior to their manufacture for clinical use. 

## Figures and Tables

**Table 1:** Highlighted studies in cellular and noncellular approaches to repairing damaged blood–CNS-barrier.

Treatment Options	Advantages	Limitations	References
***Cellular Approaches to Repairing Damaged B-CNS-B***			
**hBM34^+^ cells**	Dose–response iv study revealed that the highest cell dose improved motor function, enhanced mn survival, reduced gliosis, decreased EB permeability, and maintained astrocyte end-feet processes in G93A SOD1 mice. Transplanted cells engrafted within spinal cord capillaries.	Undifferentiated transplanted cells expressing CD45 antigen were detected within capillary lumen and at a distance from blood vessels in spinal cord.	Garbuzova-Davis et al. (2017) [47]
	High cell dose showed ultrastructural morphology improvement.	Severely damaged capillaries were still detected in spinal cord via ultrastructural analysis.	Garbuzova-Davis et al. (2018) [48]
	Significant reduction of microhemorrhages noted in the gray and white matter spinal cords of mice with mid or high cell dose treatment.	Some microhemorrhages were present in the spinal cords of control mice.	Eve et al. (2018) [49]
**hBMEPCs**	Cell iv transplantation enhanced behavioral disease outcomes and mn survival, restored capillary ultrastructure, reduced EB permeability, and re-established perivascular astrocyte end-feet in G93A SOD1 mice. Transplanted cells engrafted into capillaries of gray/white matter spinal cord and brain motor cortex/brainstem.	Ultrastructural capillary analysis was not performed, and vascular permeability was not analyzed in the brains of treated ALS mice. Post-transplant effects on TJ protein expressions were not identified.	Garbuzova-Davis et al. (2019b) [51]
	Greater levels of human DNA were detected in mouse ECs isolated from brain and spinal cord tissues of ALS mice treated with hBMEPCs vs. hBM34^+^ at the same cell dose.	ECs were isolated from a combination of brain and spinal cord tissues. Determining human DNA in ECs isolated separately from the brain and spinal cords is needed to determine post-transplant cell distribution.	Garbuzova-Davis et al. (2021a) [52]
	Isolated EC viability was higher in ALS mice receiving hBMEPCs vs. hBM34^+^ cells.		
	Significantly upregulated TJ protein expressions, improved capillary pericyte coverage, amended basement membrane laminin, and enhanced endothelial cytoskeletal F-actin were detected in spinal cord capillaries from ALS mice treated with hBMEPCs vs. hBM34+ cells at the same cell dose.	TJ proteins in segmented regions of the brain and spinal cord were not analyzed. Additional basement membrane components were not evaluated.	Garbuzova-Davis et al. (2021b) [53]
	Behavioral outcomes were ameliorated near end-stage disease and significantly increased lifespan was detected in G93A SOD1 mice receiving hBMEPCs vs. hBM34^+^ cells at the same cell dose.	The modest increase in lifespan needs to be addressed for improving the treatment’s long-term effectiveness.	Garbuzova-Davis & Borlongan (2023) [54]
**MSCs**	MSC iv administration improved behavioral motor function, reduced mn loss, decreased EB leakage, enhanced pericyte capillary coverage, and increased neurturin expression in lumbar spinal cords of treated G93A SOD1 rats.	MSCs engrafted outside of CNS endothelium. Specific cell type(s) differentiated from MSCs, contributing to BSCB repair, were not determined.	Magota et al. (2021) [59]
***Noncellular Approaches to Repairing Damaged B-CNS-B***			
**SC-derived nanovesicles**	Colony-forming EPC-derived exosomes administered iv into mice with TBI significantly reduced EB leakage and brain edema.	A modest decrease in EB extravasation was detected in treated mice.	Gao et al. (2018) [74]
	MSC-derived EVs administered iv into SCI rats improved locomotor function, reduced neuronal cell death, decreased EB leakage, and improved pericyte capillary coverage.	Direct EV interactions with damaged ECs were not determined.	Lu et al. (2019) [76]
	Repeated intranasal or iv administration of adipose MSC-derived exosomes improved motor function, increased mn survival, and decreased gliosis in G93A SOD1 mice.	Treatment was ineffective in delaying progression in late-stage disease. The effects of exosomes on the CNS endothelium were undetermined.	Bonafede et al. (2020) [78]
	hBMEPC-derived EVs dose-dependently increased mBEC survival in an ALS-like environment in vitro. Uptake of EVs into mBECs in pathological condition was established.	EVs at a dose of 5 µg/mL increased mBEC death in pathologic conditions. EV effects on endothelium repair in ALS were undetermined in vivo.	Garbuzova-Davis et al. (2020) [40]
	Human umbilical cord MSC-derived EVs delivered iv in a mouse model of ischemic stroke and tPA-induced injury model reduced hemorrhages, EB extravasation, and decreased neurological deficits.	The direct effects of EVs on ECs in the BBB were not determined.	Qiu et al. (2022) [75]
	MSC-EVs derived from human umbilical cord administered iv into SCI rats decreased BSCB permeability and increased TJ expressions in the spinal cord.	Only female rats were used.	Xue et al. (2023) [77]
**Bioengineered nanovesicles**	NV-IONPs from hMSCs incorporating IONPs significantly reached the injured SC after iv administration and magnetic guidance to damaged site, resulting in reduction of cell apoptosis and neuroinflammation, and behavioral improvement in an SCI mouse model. In vitro, NV-IONPs enhanced human umbilical vein’s EC proliferation and migration.	The trafficking of NV-IONPs to the damaged CNS endothelial barrier has not been studied in vivo. BSCB integrity with EB permeability was not determined.	Kim et al. (2018) [42]
	BM-MSC-derived exosomes transfected with CXCR4 increased angiogenesis and proliferation of ECs in vitro. In a rat ischemic model, injection of exosomes into lv improved behavioral recovery and reduced infarct volume in the brain.	CXCR4-overexpressing exosomes did not increase brain microvasculature EC migration more than control exosomes.	Li et al. (2020) [79]
	EVs derived from human dermal fibroblasts transfected with angiogenic factors promoted angiogenesis and enhanced wound healing in *nude* mice. These EVs also induced somatic cells towards EC differentiation.	CNS barrier integrity was not determined in vivo. EVs’ effects on EC status were not shown.	Rincon-Benavides et al. (2023) [83]
	Intranasal administration of RGD expressing-CD146+CD271+ human umbilical cord MSC-exosomes into mice with SCI reduced EB leakage, increased TJ protein expressions, and improved neurological recovery.	The exosome effects on ECs within brain capillaries were undetermined.	Xie et al. (2023) [84]
**Apolipoprotein A1 (ApoA1)**	ApoA1 dose-dependently reduced mBEC death in an ALS-like environment in vitro. ApoA1 integrated into mBECs.	ApoA1 effects on endothelium status in ALS were undetermined in vivo.	Garbuzova-Davis et al. (2022) [89]
**Activated protein C (APC)**	IP injection of APC into symptomatic G93A SOD1 mice slowed disease progression and extended survival. APC treatment reduced serum protein leakage, restored TJ protein expression, and delayed microglia activation.	Whether APC’s protective effects on damaged ECs in ALS are primary or secondary results from treatment should be elucidated.	Zhong et al. (2009) [97]

Abbreviations: BBB—blood–brain barrier, B-CNS-B—blood–CNS barrier, BSCB—blood–spinal cord barrier, EB—Evans blue dye, EV—extracellular vesicle, iv—intravenous, hBM34^+^ cells—human bone-marrow-derived CD34^+^ cells, hBMEPCs—human bone marrow derived endothelial progenitor cells, ip—intraperitoneal, lv—lateral ventricle, mn—motor neuron, mBECs—mouse brain endothelial cells, MSCs—mesenchymal stem cells, NV-IONPs—iron oxide nanoparticle-incorporated exosome-mimetic nanovesicles, SC—stem cell, SCI—spinal cord injury, TBI—traumatic brain injury, TJ—tight junction.
